# Simvastatin for patients with acute respiratory distress syndrome: long-term outcomes and cost-effectiveness from a randomised controlled trial

**DOI:** 10.1186/s13054-017-1695-0

**Published:** 2017-05-17

**Authors:** A. Agus, C. Hulme, R. M. Verghis, C. McDowell, C. Jackson, C. M. O’Kane, J. G. Laffey, D. F. McAuley

**Affiliations:** 1 Northern Ireland Clinical Trials Unit, Elliot Dynes Building, The Royal Hospitals, Grosvenor Road, Belfast, BT12 6BA UK; 2 Academic Unit of Health Economics, University of Leeds, Charles Thackrah Building, Clarendon Road, Leeds, LS2 9LJ UK; 3 Centre for Infection and Immunity, Queen’s University of Belfast, Belfast, BT9 7AE UK; 4 Department of Anaesthesia, School of Medicine, HRB Galway Clinical Research Facility, Clinical Sciences Institute, National University of Ireland, Galway, Ireland; 5 Department of Anesthesia, Centre for Critical Care Research, Keenan Research Centre for Biomedical Science, St. Michael’s Hospital, University of Toronto, Toronto, Canada; 6 Regional Intensive Care Unit, The Royal Hospitals, Grosvenor Road, Belfast, BT12 6BA UK

**Keywords:** Acute respiratory distress syndrome, Statins, Critical care, Cost-effectiveness, QALYs

## Abstract

**Background:**

Simvastatin therapy for patients with acute respiratory distress syndrome (ARDS) has been shown to be safe and associated with minimal adverse effects, but it does not improve clinical outcomes. The aim of this research was to report on mortality and cost-effectiveness of simvastatin in patients with ARDS at 12 months.

**Methods:**

This was a cost-utility analysis alongside a multicentre, double-blind, randomised controlled trial carried out in the UK and Ireland. Five hundred and forty intubated and mechanically ventilated patients with ARDS were randomly assigned (1:1) to receive once-daily simvastatin (at a dose of 80 mg) or identical placebo tablets enterally for up to 28 days.

**Results:**

Mortality was lower in the simvastatin group (31.8%, 95% confidence interval (CI) 26.1–37.5) compared to the placebo group (37.3%, 95% CI 31.6–43.0) at 12 months, although this was not significant. Simvastatin was associated with statistically significant quality-adjusted life year (QALY) gain (incremental QALYs 0.064, 95% CI 0.002–0.127) compared to placebo. Simvastatin was also less costly (incremental total costs –£3601, 95% CI –8061 to 859). At a willingness-to-pay threshold of £20,000 per QALY, the probability of simvastatin being cost-effective was 99%. Sensitivity analyses indicated that the results were robust to changes in methodological assumptions with the probability of cost-effectiveness never dropping below 90%.

**Conclusion:**

Simvastatin was found to be cost-effective for the treatment of ARDS, being associated with both a significant QALY gain and a cost saving. There was no significant reduction in mortality at 12 months,

**Trial registration:**

ISRCTN, 88244364. Registered 26 November 2010.

**Electronic supplementary material:**

The online version of this article (doi:10.1186/s13054-017-1695-0) contains supplementary material, which is available to authorized users.

## Background

The acute respiratory distress syndrome (ARDS) is a clinical syndrome characterised by life-threatening respiratory failure requiring mechanical ventilation. ARDS affects all age groups; it has a high mortality [1, 2] and causes a long-term reduction in quality of life for survivors [3]. ARDS has significant resource implications, prolonging intensive care unit (ICU) and hospital stay, and requiring rehabilitation in the community [4]. The average cost per ICU bed day in the UK National Health Service (NHS) exceeds £1200, and the delivery of critical care to patients with ARDS accounts for an important proportion of ICU capacity. Only 54% of survivors are able to return to work 12 months after hospital discharge [5].

The HARP-2 trial [5, 6] tested the hypothesis that treatment with enteral simvastatin 80 mg daily would improve clinical outcomes in patients with ARDS compared to placebo. The clinical findings [6] reported that simvastatin therapy was safe and associated with minimal adverse effects, but it did not improve clinical outcomes for patients with ARDS assessed in terms of ventilator-free days (VFDs), non-pulmonary organ failure-free days, and 28-day mortality. However, decisions on how resources should be allocated to maximise the health of the population are increasingly reliant on evidence of cost-effectiveness. The National Institute for Health and Care Excellence (NICE) [7] recommend that the effect on health-related quality of life (HRQoL) of an intervention is also quantified to enable the calculation of quality-adjusted life years (QALYs) and the cost per QALY.

Few studies have assessed the cost-effectiveness analyses of treatments for ARDS within the context of a randomised controlled trial [8–10]. CESAR [8] and OSCAR [9] compared conventional ventilator support with an alternative device: extracorporeal membrane oxygenation (ECMO) and high-frequency oscillation ventilation (HFOV), respectively. BALTI-2 [10] compared the impact of an intravenous infusion of salbutamol with placebo. In all cases, the within-trial cost per QALY estimates far exceeded the £20,000 threshold of NICE.

The aim of this paper was to report on the mortality and cost-effectiveness of enteral simvastatin in patients with ARDS at 12 months.

## Methods

### Study design

The HARP-2 trial has been described in detail elsewhere [6, 11]. In brief, this was a multicentre, double-blind, randomised control trial which recruited 540 adult patients from general ICUs in 40 hospitals in the UK and Ireland. Patients were eligible if they were intubated and mechanically ventilated and were within 48 h of onset of ARDS. Patients were randomly assigned (1:1) to receive once-daily simvastatin (at a dose of 80 mg) or identical placebo tablets enterally for up to 28 days. The Northern Ireland Clinical Trials Unit (NICTU) co-ordinated the overall study.

The cost-effectiveness of simvastatin compared to placebo was assessed using a cost-utility analysis (CUA) conducted alongside HARP-2 following the guidelines for health technology assessment in the UK [7]. The analysis was performed from the perspective of the National Health Service and Personal Social Services, and the health outcome used in the CUA was the QALY. The time horizon for the analysis was 12 months. The mortality for all patients at 12 months was also reported.

### Data collection

Data from Health and social care service use relating to primary hospital admission of patients were collected prospectively via the case report form until primary hospital discharge or death. Service use of patients after hospital discharge until 12 months post-randomisation was collected retrospectively via questionnaires posted out to surviving patients at 6 and 12 months. Medication use other than the study drug was not included in the economic analysis to minimise the burden of recall on patients. Mortality status was established via the research site or general practitioner for patients recruited in Northern Ireland and via NHS Digital (Reference number MR1294) for patients recruited in England, Scotland, and Wales. Individual-level resource use was combined with unit costs to estimate costs for each participant. Unit costs were obtained from publicly available sources and set at 2013–2014 prices (Table 1) [12–14, 24].

**Table 1:** Unit costs (UK £) of health service contacts

Resource item	Unit cost (£)	Source
Primary admission		
Intensive care level 1 day	696.12	NHS reference costs 2013–2014 (XC07Z) adult critical care [12]
Intensive care level 2 day	932.10	NHS reference costs 2013–2014 (XC06Z) adult critical care [12]
Intensive care level 3 day	1440.64	NHS reference costs 2013–2014 (XC01Z-XC05Z weighted average) adult critical care [12]
Other intensive care unit day	1228.65	NHS reference costs 2013–2014 (XC01Z-XC07Z weighted average) adult critical care [12]
High dependency unit day	932.10	NHS reference costs 2013–2014 (XC06Z) adult critical care [12]
Ward bed day	437.00	NHS reference costs 2013–2014 (VC40Z) rehabilitation for respiratory disorders [12]
Simvastatin 80 mg tablets 28 tabs/pack	2.02	National Health Service England and Wales (2014) NHS electronic drug tariff (online; accessed 24 June 2015) [14]
Other hospital services		
Non-specific ward days	483.04	NHS reference costs 2013–2014 (weighted average length of stay and cost of non-elective long stays ) [12]
Outpatient attendance	109.00	Unit costs of health and social care 2014 p.111 [13]
Attendance at Accident and Emergency department	233.00	Unit costs of health and social care 2014 p.111 (see and treat and convey) [13]
Community health services		
GP surgery consultation	46.00	Unit costs of health and social care 2014 p.195 [13]
GP telephone consultation	28.00	Unit costs of health and social care 2014 p.195 [13]
GP home consultation	115.00	Unit costs of health and social care 2013 p.191 (inflated using the hospital and community health services index) [24]
GP out of hours consultation	115.00	Unit costs of health and social care 2013 p.191 (home visit unit cost assumed as above) [24]
GP nurse surgery consultation	13.70	Unit costs of health and social care 2014 p.192 (per 15.5 min surgery consultation) [13]
GP nurse telephone consultation	4.85	Unit costs of health and social care 2014 p.192 (per 7.1 min telephone consultation)* [13]
GP nurse home visit	24.29	Unit costs of health and social care 2014 p.192 (per 15.5 min consultation and 12 min travel assumed*) [13]
District nurse home visit	39.00	Unit costs of health and social care 2014 p.187 [13]
Social worker visit	79.00	Unit costs of health and social care 2014 p.206 (per 1 h cost assumed to include travel) [13]
Physiotherapist visit	51.00	Unit costs of health and social care 2014 p.179 [13]
Occupational therapist visit	77.00	Unit costs of health and social care 2014 p.180 [13]
Dietician visit	37.00	Unit costs of health and social care 2014 p.238 [13]
Nurse specialist visit	74.00	Unit costs of health and social care 2014 p.190 (per 1 h cost assumed to include travel) [13]
Rapid response/ acute care episode	182.00	Unit costs of health and social care 2013 p.111 (inflated using the hospital and community health services index) [24]
Psychotherapy/counselling	50.00	Unit costs of health and social care 2014 p.51 [13]
Day centre	38.00	Unit costs of health and social care 2014 p.38 (per client session) [13]
Care services		
Home help/care worker	17.00	Unit costs of health and social care 2014 p.210 (per 1 h cost assumed to include travel) [13]
Delivered meals	6.60	Unit costs of health and social care 2014 p.127 (per meal) [13]
Nursing home	511.00	Unit costs of health and social care 2014 p.33 (per week) [13]
Respite	511.00	Nursing home cost assumed as above
Residential care home	493.00	Unit costs of health and social care 2014 p.34 (weekly) [13]
Sheltered housing	443.00	Unit costs of health and social care 2014 p.39 (extra care housing for older people, weekly) [13]

*General practitioner (GP) time estimates assumed when not available for GP nurse

*NHS* National Health Service

The HRQoL of patients was measured at discharge, and at 3, 6, and 12 months using the generic EuroQol Five Dimension (Three Level) (EQ-5D-3L) [15], and the UK social preference weights were used to obtain single utility values from the responses [16]. The EQ-5D is the NICE [7] preferred measure of HRQoL for economic evaluations and has been used previously in the critically ill [8–10]. As patients were unconscious at baseline, the utility value for an unconscious state (–0.402) was used; this was in keeping with previous economic evaluations of therapies for patients with ARDS [9, 10]. The area-under-the-curve method was used to estimate patient-specific QALYs accrued over the study period. Since patients still in hospital at 3 months were not administered the EQ-5D-3L and the timing of the discharge EQ-5D-3L varied, QALYS were calculated using only the baseline, 6-, and 12-month EQ-5D-3L values.

### Statistical analysis

The difference in mortality between groups was analysed using the risk ratio and *p* value from Fishers’ exact test. Time-to-event (death) data were presented using a Kaplan-Meier plot.

The cost-utility analysis included only patients with complete data on costs and QALYs in order to maintain the correlation structure of the data. Mean imputation was used for missing service use data in cases where the patient reported using a care service (e.g. carer, home help, delivered meals) but did not provide the number of contacts per week. Death was not considered a censoring event and periods after death were counted as observations with known outcome [17]. In practice, this meant that an EQ-5D-3L utility of zero was assigned for the time points after death. For patients who had died in hospital, costs after hospital discharge until 12 months follow-up were considered to be zero. For patients who were discharged from hospital but were dead at 28 days we also assumed their costs after hospital discharge until 12 months to be zero. This was an acceptable assumption since, of the patients who were dead at 28 days (24.5%; 132/539), only two patients were discharged from hospital and they both subsequently died within 2 weeks of discharge. For patients who were dead at 6 months, costs from 6 to 12 months were considered to be zero. In some cases we could not assign zero costs to all periods after death. This was the case for patients who were discharged from hospital, alive at 28 days but were dead at 6 months; costs from discharge to 6 months were considered to be missing as no information was available on their use of resources in the period up to their death. The same was true for patients alive at 6 months but dead at 12 months; costs from 6 to 12 months were treated as missing. Costs and QALYs were not discounted as the time horizon of the study was 12 months.

Descriptive statistics were used to summarise the health service resource use and associated costs for the primary admission, discharge to 6 months and 6 to 12 months, EQ-5D-3L utilities, and QALYs. Significance was judged where the confidence intervals (CIs) of differential means excluded zero or *p* < 0.05.

The mean difference in cost and QALYs between groups was estimated and the incremental cost-effectiveness ratio (ICER) calculated (if appropriate) to estimate the cost per QALY. Sampling uncertainty around the cost and QALY estimates was investigated using non-parametric bootstrapping. This involved re-sampling (with replacement) cost and QALY pairs from the original sample to generate 1000 replicates of mean differences in cost and QALYs. These were then plotted on the cost-effectiveness plane to display their joint distribution. The resulting scatter plot was used to derive the cost-effectiveness acceptability curve (CEAC) by calculating the proportion of the ICER replicates which would be considered cost-effective at various thresholds of willingness-to-pay (WTP) for an additional QALY. In general, NICE [18] consider interventions with an ICER of less than £20,000 to be cost-effective. All analyses were performed using Stata 12/IC for Windows®.

CEACs were also constructed for the following sensitivity analyses:

1. Multiple regression was used to estimate the mean difference between groups for total health service costs and QALYs after adjusting for the baseline variables of age, the Acute Physiology and Chronic Health Evaluation (APACHE) II score, and vasopressor requirement.
2. Missing total cost and QALY data points were filled simultaneously using multiple imputation by chained equations and predictive mean matching to generate five imputed datasets. Treatment group, baseline APACHE II score, age, vasopressor requirement at baseline, mortality at 28 days and mortality at 12 months, and primary admission costs were entered into the model as predictors of missing data.
3. Multiple imputation and adjustment for baseline variables simultaneously.
4. Death was treated as a censored event, i.e. data were considered to be missing for patients who had died over the study period.
5. Mean imputation was not used for missing care service data, i.e. treated as missing.
6. Discharge and 3-month EQ-5D-3L data were used in the calculation of QALYs where available.

All curves were constructed regardless of whether the cost and effect differences were statistically significant, in keeping with current health economic practice. Sample size was based on the primary outcome (VFDs) and not on the basis of mortality alone, costs, QALYs, or cost-effectiveness.

## Results

**Fig. 1:**
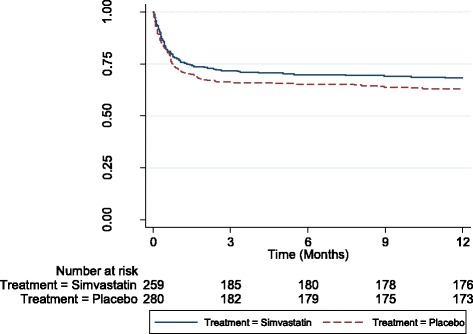
Kaplan-Meier plot for probabilities of survival over the 12-month study period according to whether patients received simvastatin or placebo

Five hundred and forty patients were randomised, 259 to receive simvastatin and 281 to receive placebo. Five patients withdrew consent over the study but only one did not give permission for the use of their anonymised data collected prior to withdrawal. Thus, 539 patients were eligible for inclusion in the analysis. Mortality was lower in the simvastatin group (31.8%, 95% CI 26.1–37.5) compared to the placebo group (37.3%, 95% CI 31.6–43.0) at 12 months, although this was not significant (*p* = 0.20). Figure 1 presents the Kaplan-Meier plot for the probabilities of survival over the study period.

**Fig. 2:**
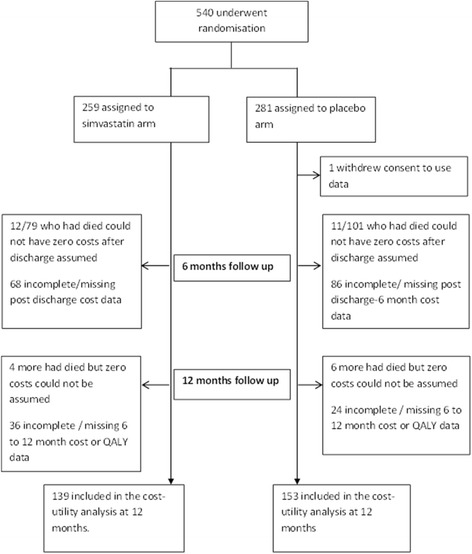
Patient drop out from the cost-utility analysis at 12 months. *QALY* quality-adjusted life year

Of the 539 patients, only 292 (54%) had complete cost and QALY data and could be included in the cost-utility analysis; 153 in the simvastatin group and 139 in the placebo. Death was not treated as a censoring event in the cost-utility analysis and so patients were included in the analysis if they had died and zero costs could be assigned as detailed in the Methods section, or if they had complete 6- and 12-month follow-up questionnaire data (Fig. 2). Baseline characteristics of patients included in the analysis were broadly similar between groups (see Additional file 1), and similar to the baseline characteristics of the original sample reported previously [6].

Patient use of health services within the categories of primary hospital admission, other hospital, community and care over the 12-month study period are presented in Additional file 2. Mean costs for these categories at 6 months and 12 months were estimated and are presented in Table 2. There was a considerable amount of variability in costs as reflected in the large standard deviations (SDs). However, lower mean costs were observed in the simvastatin group for all service categories except for other hospital services from baseline to 6 months with a total incremental cost of –£3601 indicating a cost-saving in favour of simvastatin. The cost-saving was largely driven by the higher mean number of ICU and high-dependency unit bed days in the placebo group and the high unit costs associated with them (Table 1). Post-discharge, the most notable differences in costs were associated with care-related services. The analyses of resource and costs for all patients with available data and not just those included in the cost-utility analysis are presented in Additional file 3. It is worth noting that, in the analysis of all patients, primary admission data was available for 532 patients and the difference in primary admission costs was only –£293 (see Table S6 in Additional file 3).

**Table 2:** Health services costs (UK £) over the study period by group^a^

Service costs	Simvastatin *n* = 139	Placebo *n* = 153	Difference (95% CI)^b^ simvastatin – placebo
	Mean (SD)	Mean (SD)	
Baseline to 6 months			
Primary admission	22,034.30 (14,673.67)	25,186.17 (20,202.18)	–3151.87 (–7755.42 to 565.96)
Other hospital services	641.23 (2351.29)	604.92 (3978.81)	36.31 (–814.56 to 673.96)
Community health Services	279.29 (583.75)	341.42 (1087.34)	–62.13 (–288.39 to 119.26)
Care-related services	186.71 (1065.18)	368.99 (1605.87)	–182.28 (–501.60 to 139.83)
6 to 12 months			
Other hospital services	604.81 (2387.51)	631.05 (3523.03)	–26.24 (–784.03 to 570.07)
Community health services	229.97 (739.66)	231.66 (645.46)	–1.69 (–159.67 to 151.49)
Care-related services	139.05 (1156.05)	352.05 (2353.14)	–213.00 (–678.17 to 177.77)
Total baseline to 6 months	23,141.53 (15,469.09)	26,501.51 (21,474.96)	–3359.98 (–8247.32 to 644.99)
Total 7–12 months	973.83 (3380.62)	1214.76 (5213.93)	–240.93 (–1323.17 to 634.04)
Total 12-month health service costs	24,115.36 (17,154.86)	27,716.27 (23,643.97)	–3600.91 (–8872.17 to 722.79)

^a^ Sample sizes based on all patients with available data

^b^ Confidence interval (CI) based on 1000 bootstrap resamples. Significance is judged when the confidence interval does not cross zero. Negative costs reflected cost-savings in favour of the intervention

*SD* standard deviation

### Health outcomes

The HRQoL of patients at 6 and 12 months (measured using the EQ-5D-3L) and QALYs at 12 months are presented in Table 3. All patients were assigned the same utility value at baseline (–0.402). There was little change in the HRQoL of patients in both groups from 6 to 12 months, but the HRQoL was statistically significantly higher in the simvastatin group at 6 months. The difference in QALYs (0.064) was also statistically significant. The analysis of utilities and QALYs for all patients with available data is presented in Additional file 3.

**Table 3:** Mean (SD) EQ-5D-3L utilities and QALYs, by treatment group

	Simvastatin *n* = 139	Placebo *n* = 153	Difference (95% CI) simvastatin – placebo
	Mean (SD)	Mean (SD)	
6 months utility	0.316 (0.373)	0.222 (0.348)	0.094 (0.012 to 0.175)
12 months utility	0.315 (0.375)	0.244 (0.371)	0.070 (–0.014 to 0.155)
QALYs^a^	0.136 (0.274)	0.072 (0.262)	0.064 (0.002 to 0.127)

^a^ Calculated using baseline (–0.402, 6-, and 12-month utilities)

*CI* confidence interval, *EQ-5D-3L* EuroQol Five Dimension (Three Level), *QALY* quality-adjusted life year, *SD* standard deviation

**Fig. 3:**
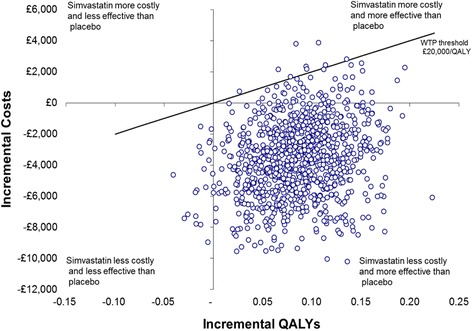
Cost-effectiveness plane for the primary cost-effectiveness analysis showing bootstrapped replications of mean incremental costs and quality-adjusted life year (*QALY*) gain and the willingness-to-pay (*WTP*) threshold of £20,000/QALY

**Fig. 4:**
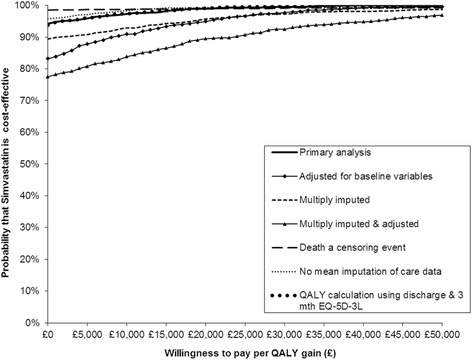
Cost-effectiveness acceptability curves showing the probability of simvastatin being cost-effective compared to placebo for the primary and sensitivity analyses. *EQ-5D-3L* EuroQol Five Dimension (Three Level), *QALY* quality-adjusted life year

Results from the primary cost-utility analysis and the sensitivity analyses are presented in Table 4. Since simvastatin was both less costly and significantly more effective than the placebo (in terms of QALYs) it can be considered the dominant strategy. In this situation the ICER would be negative and is therefore not calculated as its magnitude does not convey any meaning [17]. Sampling uncertainty in the data is represented by the joint distribution of the bootstrapped differences in cost and QALY on the cost-effectiveness plane for the primary analysis (Fig. 3). The majority of the points lie below the *x* axis indicating simvastatin is cost saving, and to the right of the *y* axis indicating simvastatin produces more QALYs than placebo. The small number of points lying outside of this area indicates a small degree of variability surrounding the presence and magnitude of cost-savings and effectiveness. The CEAC for the primary analysis presented in Fig. 4 summarises this uncertainty for the decision maker and presents the probability of simvastatin being cost-effective compared to placebo at different thresholds of WTP per QALY gain for the primary and sensitivity analyses. The CEAC for the primary analysis indicates that, at a WTP threshold of £20,000 per QALY gain, the probability of simvastatin being cost-effective is 99%.

**Table 4:** Incremental costs and QALYs (with 95% CI), associated incremental cost-effectiveness ratios, and the probability of simvastatin being cost-effective compared to placebo at a threshold willingness to pay/QALY of £20,000 for the base case and sensitivity analyses

Analysis	Incremental total health service costs (UK £; 95% CI^a^)	Incremental QALY gain (95% CI^a^)	Probability of cost-effectiveness at £20,000 per QALY (%)
Primary analysis (unadjusted) (simvastatin *n* = 139, placebo *n* = 153^b^)	–3600.91 (–8061.10 to 859.28)	0.064 (0.002 to 0.127)	99%
Adjusted for baseline variables (simvastatin *n* = 139, placebo *n* = 153^b^)	–2661.03 (–7842.76 to 2520.70)	0.089 (0.025 to 0.151)	95%
Multiply imputed total costs and QALYs (simvastatin *n* = 259, placebo *n* = 280^c^)	–2132.69 (–5629.21 to 1363.83)	0.042 (–0.001 to 0.086)	96%
Multiply imputed total costs and QALY, adjusted (simvastatin *n* = 259, placebo *n* = 280^c^)	–1290.35 (–5000.61 to 2419.91)	0.048 (0.005 to 0.091)	90%
Death as a censoring event (simvastatin *n* = 74, placebo *n* = 68^b^)	–8532.48 (–16107.75 to –957.21)	0.056 (–0.022 to 0.135)	99%
No mean imputation of care data (simvastatin *n* = 137, placebo *n* = 151^b^)	–3966.00 (–8503.11 to 571.10)	0.066 (0.004 to 0.128)	99%
QALY calculation using discharge, 3-, 6-, and 12-month EQ-5D-3 L (simvastatin *n* = 138, placebo *n* = 150^b^)	–3559.00 (–8241.41 to 1123.42)	0.084 (0.005 to 0.162)	99%

^a^ Confidence intervals (CI) based on 1000 bootstrap resamples

^b^ Sample sizes based on patients with complete data for both costs and quality-adjusted life years (QALYs)

^c^ Sample sizes based on all patients since missing total costs and QALYs have been imputed

*EQ-5D-3L* EuroQol Five Dimension (Three Level)

Sensitivity analyses were conducted to determine the impact of changing particular assumptions on the cost-effectiveness (Table 4). Although there are some notable effects on differential mean costs and effects, the CEACs for the sensitivity analyses Fig. 4 indicate that cost-effectiveness of simvastatin was robust to these changes in the assumptions, with the probability of it being cost-effective at £20,000 per QALY never dropping below 90%.

## Discussion

The results of the cost-utility analysis alongside the HARP-2 trial indicate that simvastatin compared to placebo was associated with lower costs and a significant QALY gain. Whilst the gain in QALYs was small (0.064; equivalent to 23 days of full health), it was associated with a cost saving equating to £3601 over the 12-month period. Simvastatin has a very high probability of being considered cost-effective at 1 year and findings were robust to changes in the methodological assumptions.

There is currently no consensus on what constitutes a minimally important difference in mean QALYs between groups [19]; however, 0.05 has been suggested previously [19, 20]. The difference observed in our analysis exceeds this, suggesting the small difference is still meaningful. The gain in QALYs corroborates with the modest benefits observed in the clinical effectiveness analysis [6]. Since all patients were assigned the same utility score at baseline, the difference in QALYs is due to the HRQoL of simvastatin patients being higher than placebo at both 6 and 12 months.

The majority of differences in individual resource use components were not statistically significant. However, when costs were categorised as primary admission, other hospital services, community health services, and care-related services, differences were more apparent. The largest difference in costs was related to patient stay in primary admission and this was driven by the high cost of ICU care. The majority of costs after discharge were lower in the simvastatin group, with the most notable cost savings associated with the primary admission. The results corroborate with the HRQoL analysis; patients in the placebo group experienced poorer health over the study period and this appears to have impacted on their use of health services after discharge

There were a number of limitations to the economic evaluation. The study was powered to detect statistically significant differences in the primary outcome and not in costs, QALYs, or cost-effectiveness. However, this is typically the case and significance rules are not typically relied upon in the interpretation of cost-effectiveness analyses [21] as greater emphasis is placed on the joint distribution of cost and effects. Nonetheless, having a sufficiently powered study would have led to more conclusive results [19] and allowed decision-makers to be more confident in the value claim [17]. There are limitations to this study related to the inevitable difficulties of collecting follow-up data from patients recovering from a stay in critical care. Previous intensive care trials [9, 10] have also found it difficult to achieve high rates of long-term data collection and the experience of HARP-2 confirms that this is a difficult population to follow-up. Furthermore, economic data are particularly prone to missing data due to the reliance on multiple components within HRQoL and resource use questionnaires required for the calculation of QALYs and costs. As a result, the cost-utility analysis was performed on a subgroup of patients with complete cost and QALY data. Although the baseline characteristics of the subgroup were observed to be similar between study groups and similar to the original sample, the impact of this on primary admission costs was notable; the mean difference increasing from –£293 when all available data were used (*n* = 532) to –£3152 for the subgroup (*n* = 292). This suggests that some of the patients who were not included in the cost-utility analysis were simvastatin patients who had incurred high primary admission costs. It is important to highlight, however, that multiple imputation of missing data was performed using primary admission costs as one of the predictors of missingness, and the cost-effectiveness of simvastatin remained high.

The utilities derived from the discharge and 3-month EQ-5D-3L were not used in the QALY calculation for the primary analysis. This was due to the variable timing of the discharge questionnaire and the 3-month questionnaire not being consistently administered to survivors if they were still hospital in-patients. A sensitivity analysis included them in the QALY calculation when they were available and there was minimal impact on the overall results. The probability of simvastatin being cost-effectiveness at a WTP of £20,000 remained at 99%.

A key strength of this study is the successful long-term follow-up of patients who have been discharged from critical care to assess their survival, HRQoL, and resource use. The results highlight the importance of undertaking a health economic analysis in the setting where the primary clinical outcome is not significantly different between the trial arms. In addition, it flags important issues regarding the use of short-term clinical outcomes such as VFDs which have been shown to poorly correlate with long-term patient-centred outcomes such as long-term mortality [22] and QALYs [22, 23]. HARP-2 achieved a non-significant 5% reduction in mortality, and the cost-utility analysis found a significant QALY gain with a non-significant cost saving at 12 months, but the trial is considered a negative trial due to the absence of a significant difference in the primary outcome (VFDs) at 28 days. In the setting of no significant difference in mortality or other clinical outcomes it is unlikely that the results of the cost-effectiveness analysis will be sufficient to change clinical practice. Had the trial been powered sufficiently for long-term mortality or QALYs a different conclusion may have been reached. QALYs may be a feasible patient-centred primary outcome for critical care studies as they combine both morbidity and mortality, and have potential gains in statistical power due to being a continuous variable [20].

## Conclusions

Simvastatin was found to be cost-effective at 1 year compared to placebo for the treatment of ARDS, being associated with both a significant QALY gain and cost saving. The cost-effectiveness remained robust to changes in methodological assumptions. However, given that the health economic analysis was performed on a subgroup of patients and the QALY gain was relatively small, there are currently insufficient data to support the treatment of patients with ARDS with simvastatin in the NHS.

## Additional files

Additional file 1: Baseline characteristics of patients included in the cost-utility analysis. (DOCX 17 kb)

Additional file 2: Patient use of health services within the categories of primary hospital admission, other hospital, community, and care over the 12-month study period for those patients with complete cost and QALY data included in the cost-utility analysis. (DOCX 29 kb)

Additional file 3: Analysis of health-related quality of life, health service use, and costs over the 12-month study period for all patients with data available, i.e. not just those patients with complete cost and QALY data used in the cost-utility analysis. (DOCX 33 kb)

Additional file 4: HARP-2 Study Group. (DOCX 22 kb)

## Abbreviations

APACHE: Acute Physiology and Chronic Health Evaluation
ARDS: Acute respiratory distress syndrome
CEAC: Cost-effectiveness acceptability curve
CI: Confidence interval
CUA: Cost-utility analysis
EQ-5D-3L: EuroQol Five Dimension (Three Level)
HRQoL: Health-related quality of life
ICER: Incremental cost-effectiveness ratio
ICU: Intensive care unit
NHS: National Health Service
NICE: National Institute for Health and Care Excellence
QALY: Quality-adjusted life year
SD: Standard deviation
VFD: Ventilator-free day
WTP: Willingness-to-pay

## References

[1] Rubenfeld GD, Caldwell E, Peabody E, Weaver J, Martin DP, Neff M (2005). Incidence and outcomes of acute lung injury. N Engl J Med.

[2] Brun-Buisson C, Minelli C, Bertolini G, Brazzi L, Pimentel J, Lewandowski K (2004). Epidemiology and outcome of acute lung injury in European intensive care units. Results from the ALIVE study. Intensive Care Med.

[3] Dowdy DW, Eid MP, Dennison CR, Mendez-Tellez PA, Herridge MS, Guallar E (2006). Quality of life after acute respiratory distress syndrome: a meta-analysis. Intensive Care Med.

[4] Rossi C, Simini B, Brazzi L, Rossi G, Radrizzani D, Iapichino G (2006). Variable costs of ICU patients: a multicenter prospective study. Intensive Care Med.

[5] Cheung AM, Tansey CM, Tomlinson G, Diaz-Granados N, Matte A, Barr A (2006). Two-year outcomes, health care use, and costs of survivors of acute respiratory distress syndrome. Am J Respir Crit Care Med.

[6] McAuley DF, Laffey JG, O'Kane CM, Perkins GD, Mullan B, Trinder TJ (2014). Simvastatin in the acute respiratory distress syndrome. N Engl J Med..

[7] National Institute for Health and Care Excellence (2013). Guide to the methods of technology appraisal 2013.

[8] Peek GJ, Elbourne D, Mugford M, Tiruvoipati R, Wilson A, Allen E, Clemens F, Firmin R, Hardy P, Hibbert C, Jones N, Killer H, Thalanany M, Truesdale A (2010). Randomised controlled trial and parallel economic evaluation of conventional ventilatory support versus extracorporeal membrane oxygenation for severe adult respiratory failure (CESAR). Health Technol Assess.

[9] Lall R, Hamilton P, Young D, Hulme C, Hall P, Shah S (2015). A randomised controlled trial and cost-effectiveness analysis of high-frequency oscillatory ventilation against conventional artificial ventilation for adults with acute respiratory distress syndrome. The OSCAR (OSCillation in ARDS) study. Health Technol Assess.

[10] Gates S, Perkins GD, Lamb SE, Kelly C, Thickett DR, Young JD, et al. Beta-Agonist Lung injury TrIal-2 (BALTI-2): a multicentre, randomised, double-blind, placebo-controlled trial and economic evaluation of intravenous infusion of salbutamol versus placebo in patients with acute respiratory distress syndrome. Health Technol Assess. 2013;17(38):1–87. 10.3310/hta17380 PMC4781532 24028755

[11] McAuley DF, Laffey JG, O’Kane CM (2012). Hydroxymethylglutaryl-CoA reductase inhibition with simvastatin in acute lung injury to reduce pulmonary dysfunction (HARP-2) trial: study protocol for a randomized controlled trial. Trials..

[12] Department of Health (2014). NHS Reference Costs 2013–14.

[13] Curtis L, editor. Unit costs of health and social care 2014. Canterbury: University of Kent: Personal Social Services Research Unit; 2014.

[14] National Health Service Electronic Drug Tariff for England and Wales. January 2014. Compiled on behalf of the Department of Health by the NHS Business Services Authority, NHS Prescription Services. http://www.ppa.org.uk/edt/January_2014/mindex.htm. Accessed 11 May 2017.

[15] EuroQol Group (1990). Euroqol – a new facility for the measurement of health-related quality of life. Health Policy..

[16] Dolan P (1997). Modelling valuations for EuroQol health states. Med Care..

[17] Glick HA, Doshi JA, Sonnad SS, Polsky D (2007). Economic evaluation in clinical trials.

[18] National Institute for Health and Clinical Excellence (2008). Social value judgements: principles for the development of NICE guidance.

[19] Hollingworth W, McKell-Redwood D, Hampson L, Metcalfe C (2013). Cost-utility analysis conducted alongside randomized controlled trials: are economic end points considered in sample size calculations and does it matter?. Clin Trials.

[20] Ferguson ND, Scales DC, Pinto R, Wilcox ME, Cook DJ, Guyatt GH, Schünemann HJ, Marshall JC, Herridge MS, Meade MO, Canadian Critical Care Trials Group (2013). Integrating mortality and morbidity outcomes: using quality-adjusted life years in critical care trials. Am J Respir Crit Care Med.

[21] Claxton K (1999). The irrelevance of inference: a decision-making approach to the stochastic evaluation of health care technologies. J Health Econ.

[22] Karir V, Angus DC, Clermont G, Kong L, Bernard GR, Rubenfeld GD, Thompson BT, Kahn JM. Relationship between ventilator-free-days and patient-centered outcomes in patients with acute lung injury. San Francisco, CA: Poster session presented at: Clinical Trials in the Intensive Care Unit, American Thoracic Society International Conference; 2012.

[23] Spragg RG, Bernard GR, Checkley W, Curtis JR, Gajic O, Guyatt G, Hall J, Israel E, Jain M, Needham DM, Randolph AG, Rubenfeld GD, Schoenfeld D, Thompson BT, Ware LB, Young D, Harabin AL (2010). Beyond mortality: future clinical research in acute lung injury. Am J Respir Crit Care Med.

[24] Curtis L, ed. Unit Costs of Health and Social Care 2013. Canterbury: University of Kent: Personal Social Services Research Unit; 2013.

