# Supplementary material for: Simvastatin for patients with acute respiratory distress syndrome: long-term outcomes and cost-effectiveness from a randomised controlled trial
Source: Crit Care. 2017 May 17;21:108. doi: 10.1186/s13054-017-1695-0 (PMC5434552; doi:10.1186/s13054-017-1695-0)
Supplement: Supplementary file 1 — Baseline characteristics of patients included in the cost-utility analysis. (DOCX 17 kb) [file 13054_2017_1695_MOESM1_ESM.docx]

**Additional File 1:**

**Simvastatin for patients with Acute Respiratory Distress Syndrome: long term outcomes and cost-effectiveness from a randomised controlled trial**

A. Agus Ph.D, C. Hulme Ph.D, R.M. Verghis M.Sc, C. McDowell M.Sc, C. Jackson C.M. O’Kane Ph.D, J.G. Laffey M.D., D.F. McAuley M.D.

Baseline characteristics of patients included in the cost-utility analysis

|  | **Simvastatin**  **n = 139** | **Placebo**  **n = 153** |
| --- | --- | --- |
| Age (years) | 56.5 (16.4) | 56.7 (16.8) |
| Gender Male | 69 (49.6) | 90 (58.8) |
| Female | 70 (50.4) | 63 (41.2) |
| Sepsis | 107 (77.0) | 125 (81.7) |
| Non Sepsis | 32 (23.0 ) | 28 (18.3) |
| Vasopressor Requirement Yes | 95 (68.4) | 107 (69.9) |
| No | 44 (31.6) | 46 (30.1) |
| Plateau Pressure (cmH_2_O) | 24.3 (6.8)  n=73 | 24.1 (6.1)  n=85 |
| APACHE II score | 20.9 (6.7)  n=121 | 19.0 (6.1)  n=137 |
| PaO_2_:FiO_2_ ratio mmHg | 117.8 (52.5) | 130.9 (54.7) |
| Tidal Volume per Ideal Body Weight (ml/kg) | 8.0 (3.0)  n=124 | 8.2 (2.8)  n=141 |
| Aetiology of ARDS |  |  |
| Direct |  |  |
| Smoke/toxin inhalation | 0 (0.0) | 1 (0.7) |
| Gastric content aspiration | 14 (10.1) | 11 (7.2) |
| Near drowning | 0 (0.0) | 0 (0.0) |
| Thoracic trauma | 11 (7.9) | 2 (1.3) |
| Pneumonia | 88 (63.3) | 78 (51.0) |
| Other | 8 (5.8) | 13 (8.5) |
| Indirect |  |  |
| Sepsis | 60 (43.2) | 70 (45.8) |
| Cardiopulmonary bypass | 1 (0.7) | 0 (0.0) |
| Pancreatitis | 2 (1.4) | 15 (9.8) |
| Non-thoracic trauma | 1 (0.7) | 4 (2.6) |
| Other | 6 (4.3) | 12 (7.8) |
| SOFA Score | 8.6 (3.2)  n=114 | 9.1 (2.8)  n=136 |
| Oxygenation Index cm of water/mm Hg | 16.4 (13.3)  n=106 | 16.6 (13.7)  n=116 |
| Lowest Mean Arterial Pressure (mmHg) | 64.6 (7.7) | 64.6 (8.8) |

Summarized as Mean (SD), n (%)

n= presented for each arm where it differs from n=139 in Simvastatin arm and n=153 in placebo arm.

#Patients can have both direct and indirect aetiology of ARDS (%s calculated over no of patient in each arm)
