# Supplementary material for: Simvastatin for patients with acute respiratory distress syndrome: long-term outcomes and cost-effectiveness from a randomised controlled trial
Source: Crit Care. 2017 May 17;21:108. doi: 10.1186/s13054-017-1695-0 (PMC5434552; doi:10.1186/s13054-017-1695-0)
Supplement: Supplementary file 2 — Patient use of health services within the categories of primary hospital admission, other hospital, community, and care over the 12-month study period for those patients with complete cost and QALY data included in the cost-utility analysis. (DOCX 29 kb) [file 13054_2017_1695_MOESM2_ESM.docx]

**Additional File 2:**

**Simvastatin for patients with Acute Respiratory Distress Syndrome: long term outcomes and cost-effectiveness from a randomised controlled trial**

A. Agus Ph.D, C. Hulme Ph.D, R.M. Verghis M.Sc, C. McDowell M.Sc, C. Jackson C.M. O’Kane Ph.D, J.G. Laffey M.D., D.F. McAuley M.D.

Reporting of patients’ use of health services within the categories of primary hospital admission, other hospital, community and care over the 12 month study period for those patients with complete cost and QALY data included in the cost-utility analysis.

Table 1 Primary admission health service use by treatment arm.

|  | **Simvastatin**  **(n=139)** | **Placebo**  **(n=153)** |  | |
| --- | --- | --- | --- | --- |
|  | **Mean (SD)** | **Mean (SD)** | **p-value** |  |
| **Primary admission (baseline to discharge)** |  |  |  |  |
| Primary ICU stay days | 14.07 (10.83) | 14.62 (11.58) | 0.677 |  |
| Intensive Care Level 1 days | 0.42 (1.22) | 0.39 (1.24) | 0.822 |  |
| Intensive Care Level 2 days | 1.56 (2.15) | 1.64 (2.54) | 0.775 |  |
| Intensive Care Level 3 days | 10.96 (7.46) | 11.34 (8.36) | 0.681 |  |
| Other ICU days^a^ | 0.40 (2.20) | 1.40 (7.27) | 0.119 |  |
| HDU days^b^ | 0.27 (1.20) | 1.41 (5.95) | 0.027 |  |
| Ward days | 8.61 (14.16) | 9.18 (16.25) | 0.750 |  |
| Simvastatin 80mg tablets | 18.34 (13.96) | - | - |  |

^a^ level of care was not recorded for days spent in ICU after 28 days, these are presented separately

^b^ some units had separate HDU units, but not all.

Table 2 Other hospital service use from baseline until 12 months by group. Values are number (percentages) of patients using the service and mean (SD) use.

|  | **Discharge – 6 months** | | | | | **6 – 12 months** | | | | |
| --- | --- | --- | --- | --- | --- | --- | --- | --- | --- | --- |
|  | **Simvastatin**  **(n=139)** | | **Placebo**  **(n=153)** | |  | **Simvastatin**  **(n=139)** | | **Placebo**  **(n=153)** | |  |
|  | **Number (%)** | **Mean (SD)** | **Number (%)** | **Mean (SD)** | **p-value** | **Number (%)** | **Mean (SD)** | **Number (%)** | **Mean (SD)** | **p-value** |
| Hospital inpatient days | 13 (9.4) | 0.81 (4.21) | 13 (8.5) | 1.02 (8.10) | 0.788 | 15 (10.8) | 0.95 (4.72) | 14 (9.2) | 1.05 (6.74) | 0.881 |
| Hospital outpatient appointment | 51 (36.7) | 1.73 (6.12) | 36 (23.5) | 0.85 (2.22) | 0.099 | 41 (29.5) | 0.97 (2.30) | 31 (20.3) | 0.79 (2.85) | 0.555 |
| Hospital accident and emergency visit | 15 (10.8) | 0.26 (1.37) | 11 (7.2) | 0.08 (0.32) | 0.128 | 13 (9.4) | 0.17 (0.70) | 13 (8.5) | 0.16 (0.63) | 0.839 |

Table 3 Community health service use from baseline until 12 months by group. Values are number (percentages) of patients using the service and mean (SD) use.

|  | **Discharge – 6 months** | | | | | **6– 12 months** | | | | |
| --- | --- | --- | --- | --- | --- | --- | --- | --- | --- | --- |
|  | **Simvastatin**  **(n=139)** | | **Placebo**  **(n=153)** | |  | **Simvastatin**  **(n=139)** | | **Placebo**  **(n=153)** | |  |
|  | **Number (%)** | **Mean (SD)** | **Number (%)** | **Mean (SD)** | **p-value** | **Number (%)** | **Mean (SD)** | **Number (%)** | **Mean (SD)** | **p-value** |
| GP surgery consultation | 54 (38.9) | 2.08 (4.27) | 46 (30.1) | 1.77 (4.59) | 0.546 | 52 (37.4) | 2.23 (6.88) | 48 (31.37) | 1.35 (2.85) | 0.142 |
| GP telephone consultation | 19 (13.7) | 0.55 (2.27) | 17 (11.1) | 0.47 (2.10) | 0.766 | 13 (9.5) | 1.02 (8.88) | 11(7.19) | 0.37 (2.13) | 0.381 |
| GP home consultation | 10 (7.2) | 0.08 (0.29) | 16 (10.5) | 0.22 (0.77) | 0.041 | 7 (5.0) | 0.09 (0.46) | 10 (6.54) | 0.14 (0.66) | 0.384 |
| GP out of hours consultation | 4 (2.9) | 0.17 (1.70) | 7 (4.6) | 0.07 (0.37) | 0.447 | 8 (5.8) | 0.06 (0.23) | 5 (3.27) | 0.05 (0.27) | 0.688 |
| GP Nurse surgery consultation | 27 (19.4) | 0.74 (2.47) | 22 (14.4) | 1.02 (4.82) | 0.541 | 29 (20.9) | 0.60 (1.76) | 16 (10.46) | 1.12 (8.19) | 0.458 |
| GP Nurse telephone consultation | 3 (2.2) | 0.14 (1.29) | 1 (0.7) | 0.01 (0.16) | 0.242 | 2 (1.4) | 0.07 (0.64) | - | - | 0.168 |
| GP Nurse home consultation | 8 (5.8) | 0.34 (1.76) | 11 (7.2) | 1.41 (11.68) | 0.284 | 2 (1.4) | 0.02 (0.19_ | 6 (3.92) | 1.03 (8.74) | 0.177 |
| District Nurse | 1 (0.7) | 0.02 (0.25) | 4 (2.6) | 0.25 (1.88) | 0.159 | 1 (0.7) | 0.17 (2.03) | 2 (1.31) | 0.05 (0.51) | 0.480 |
| Social Worker | 6 (4.3) | 0.12 (0.76) | 7 (4.6) | 0.08 (0.43) | 0.607 | 3 (2.2) | 0.08 (0.70) | 7 (4.58) | 0.09 (0.55) | 0.867 |
| Physiotherapist | 25 (18.0) | 1.43 (4.82) | 17 (11.1) | 1.05 (6.22) | 0.564 | 13 (9.4) | 0.47 (1.94) | 14 (9.15) | 0.98 (5.37) | 0.288 |
| Occupational Therapist | 13 (9.4) | 0.39 (2.21) | 8 (5.2) | 0.90 (9.16) | 0.526 | 8 (5.8) | 0.40 (2.99_ | 8 (5.23) | 0.25 (1.74) | 0.602 |
| Dietician | 2 (1.4) | 0.03 (0.27) | 1 (0.7) | 0.02 (0.24) | 0.759 | - | - | - | - |  |
| Nurse Specialist | 1 (0.7) | 0.02 (0.25) | - | - | 0.295 | - | - | - | - |  |
| Rapid Response | 3 (2.2) | 0.02 (0.15) | 3 (2.0) | 0.14 (1.54) | 0.378 | 3 (2.2) | 0.02 (0.15) | 2 (1.31) | 0.02 (0.18) | 0.917 |
| Psychotherapy/ counselling | - | - | 1 (0.7) | 0.01 (0.16) | 0.341 | 1 (0.7) | 0.01 (0.08) | 1 (0.65) | 0.29 (3.64) | 0.353 |
| Day centre | - | - | - | - | - | 2 (1.4) | 0.13 (1.44) | - | - | 0.268 |

Table 4 Care service use from baseline until 12 months by group. Values are number (percentages) of patients using the service and mean (SD) use.

| **Service** | **Discharge – 6 months** | | | | | **6– 12 months** | | | | |
| --- | --- | --- | --- | --- | --- | --- | --- | --- | --- | --- |
|  | **Simvastatin (n=139)** | | **Placebo (n=153)** | |  | **Simvastatin (n=139)** | | **Placebo (n=153)** | |  |
|  | **Number (%)** | **Mean (SD)** | **Number (%)** | **Mean (SD)** | **p-value** | **Number (%)** | **Mean (SD)** | **Number (%)** | **Mean (SD)** | **p-value** |
| Home Help visits | - | - | 3 (2.0) | 2.34 (17.01) | 0.107 | - | - | 1 (0.65) | 4.76 (58.86) | 0.341 |
| Meals on Wheels visits | - | - | - | - | - | - | - | 1 (0.65) | 0.68 (8.41) | 0.341 |
| Carer visits (paid by NHS) | 4 (2.9) | 4.00 (24.74) | 7 (4.6) | 13.90 (80.62) | 0.166 | 3 (2.16) |  | 6 (3.92) | 15.29 (91.03) | 0.108 |
| Residential Care Home (days) | - | - | - | - | - | - | - | - | - | - |
| Nursing Home (days) | 3 (2.2) | 1.45 (13.07) | 2 (1.3) | 0.42 (3.71 | 0.352 | 1 (0.72) | 1.29 (15.27) | - | - | 0.295 |
| Shelter (days) | - | - | 1 (0.7) | 0.98 (12.13) | 0.341 | - | - | - | - | - |
| Respite Care (days) | 1 (0.7) | 0.17 (2.04) | - | - | 0.295 | - | - | 1 (0.65) | 0.09 (1.13) | 0.341 |
