# Supplementary material for: Simvastatin for patients with acute respiratory distress syndrome: long-term outcomes and cost-effectiveness from a randomised controlled trial
Source: Crit Care. 2017 May 17;21:108. doi: 10.1186/s13054-017-1695-0 (PMC5434552; doi:10.1186/s13054-017-1695-0)
Supplement: Supplementary file 3 — Analysis of health-related quality of life, health service use, and costs over the 12-month study period for all patients with data available, i.e. not just those patients with complete cost and QALY data used in the cost-utility analysis. (DOCX 33 kb) [file 13054_2017_1695_MOESM3_ESM.docx]

**Additional File 3:**

**Simvastatin for patients with Acute Respiratory Distress Syndrome: long term outcomes and cost-effectiveness from a randomised controlled trial**

A. Agus Ph.D., C. Hulme Ph.D., R.M. Verghis M.Sc., C. McDowell M.Sc., C. Jackson, C.M., O’Kane Ph.D., J.G. Laffey M.D., D.F. McAuley M.D.

Analysis of health-related quality of life, health service use and costs over the 12 month study period for **all patients with data available** i.e. not just those patients with complete cost and QALY data used in the cost-utility analysis.

Table S1 EQ-5D-3L utilities, by treatment arm.^a^

|  | **Simvastatin** | | **Placebo** | | **p-value** |
| --- | --- | --- | --- | --- | --- |
|  | **n** | **Mean (SD)** | **n** | **Mean (SD)** |  |
| **EQ-5-3L utilities** |  |  |  |  |  |
| Discharge^b^ | 128 | 0·46 (0·38) | 129 | 0·44 (0·37) | 0·630 |
| 3 months | 182 | 0·28 (0·38) | 194 | 0·25 (0·37) | 0·457 |
| 6 months | 184 | 0·31 (0·38) | 188 | 0·23 (0·36) | 0·059 |
| 12 months | 167 | 0·29 (0·37) | 193 | 0·24 (0·37) | 0·143 |

a. Sample sizes based on all patients with available data.

b Sample size is lower due to it only be relevant to patients who were discharged (i.e. did not die in hospital). For the other time points the sample size includes patients who had died.

SD; standard deviation

Table S2 Primary admission health service use by treatment arm.^a^

|  | **Simvastatin** | | **Placebo** | |  |
| --- | --- | --- | --- | --- | --- |
|  | **n** | **Mean (SD)** | **n** | **Mean (SD)** | **p-value** |
| **Primary admission (baseline to discharge)** |  |  |  |  |  |
| Primary ICU stay days | 259 | 15 (13·41) | 280 | 15 (12·5) | 0·997 |
| Intensive Care Level 1 days | 259 | 0·53 (1·2) | 280 | 0·46 (1·14) | 0·451 |
| Intensive Care Level 2 days | 259 | 2·32 (2·85) | 280 | 2·31 (3·1) | 0·958 |
| Intensive Care Level 3 days | 259 | 10·66 (7·56) | 280 | 10·77 (8·24) | 0·866 |
| Other ICU days^b,c^ | 257 | 1·00 (4·84) | 275 | 1·19 (6·33) | 0·695 |
| HDU days^b^ | 257 | 0·82 (4·28) | 275 | 1·75 (8·05) | 0·098 |
| Ward days^b^ | 257 | 13·91 (26·10) | 275 | 12·21 (18·61) | 0·285 |
| Simvastatin 80mg tablets | 259 | 19·93 (14·24) | - | - | - |

a sample sizes based on all patients with available data.

b 7 patients had unobtainable hospital discharge dates

c level of care was not recorded for days spent in ICU after 28 days, these are presented separately

Table S3 Other hospital service use from baseline until 12 months by group. Values are number (percentages) of patients using the service and mean (SD) use. ^a^

|  | **Discharge – 6 months** | | | | | **6 – 12 months** | | | | |
| --- | --- | --- | --- | --- | --- | --- | --- | --- | --- | --- |
|  | **Simvastatin**  **(n=179)** | | **Placebo**  **(n=184)** | |  | **Simvastatin**  **(n=168)** | | **Placebo**  **(n=190)** | |  |
|  | **Number (%)** | **Mean (SD)** | **Number (%)** | **Mean (SD)** | **p-value** | **Number (%)** | **Mean (SD)** | **Number (%)** | **Mean (SD)** | **p-value** |
| Hospital inpatient days | 18 (10·06) | 0·99 (4·72) | 21 (11·41) | 1·28 (7·92) | 0·675 | 18 (10·71) | 0·89 (4·36) | 20 (10·53) | 0·99 (6·15) | 0·857 |
| Hospital outpatient appointment | 73 (40·78) | 1·72 (5·49) | 54 (29·35) | 1·36 (4·86) | 0·506 | 51 (30·36) | 1·03 (2·29) | 43 (22·63) | 0·84 (2·68) | 0·467 |
| Hospital accident and emergency visit | 22 (12·29) | 0·30 (1·43) | 17 (9·24) | 0·12 (0·40) | 0·097 | 17 (10·12) | 0·17 (0·65) | 16 (8·42) | 0·15 (0·59) | 0·769 |

a sample sizes based on all patients with available data.

Table S4 Community health service use from baseline until 12 months by group. Values are number (percentages) of patients using the service and mean (SD) use. ^a^

|  | **Discharge – 6 months** | | | | | **6– 12 months** | | | | |
| --- | --- | --- | --- | --- | --- | --- | --- | --- | --- | --- |
|  | **Simvastatin**  **(n=179)** | | **Placebo**  **(n=184)** | |  | **Simvastatin**  **(n=168)** | | **Placebo**  **(n=190)** | |  |
|  | **Number (%)** | **Mean (SD)** | **Number (%)** | **Mean (SD)** | **p-value** | **Number (%)** | **Mean (SD)** | **Number (%)** | **Mean (SD)** | **p-value** |
| GP surgery consultation | 79 (44·13) | 2·41 (4·57) | 65 (35·33) | 2 (4·46) | 0·383 | 61 (36·31) | 2·11 (6·35) | 62 (32·63) | 1·42 (2·84) | 0·176 |
| GP telephone consultation | 28 (15·64) | 0·72 (2·98) | 25 (13·59) | 0·74 (3·1) | 0·927 | 18 (10·71) | 0·93 (8·09) | 17 (8·95) | 0·44 (2·09) | 0·414 |
| GP home consultation | 19 (10·61) | 0·26 (1·45) | 23 (12·5) | 0·33 (1·29) | 0·66 | 9 (5·36) | 0·11 (0·54) | 17 (8·95) | 0·23 (0·93) | 0·149 |
| GP out of hours consultation | 6 (3·35) | 0·15 (1·51) | 10 (5·43) | 0·07 (0·33) | 0·513 | 11 (6·55) | 0·08 (0·31) | 9 (4·74) | 0·08 (0·41) | 0·968 |
| GP Nurse surgery consultation | 34 (18·99) | 0·73 (2·56) | 29 (15·76) | 1·22 (5·3) | 0·27 | 32 (19·05) | 0·57 (1·74) | 18 (9·47) | 0·93 (7·36) | 0·542 |
| GP Nurse telephone consultation | 3 (1·68) | 0·11 (1·14) | 2 (1·09) | 0·05 (0·61) | 0·59 | 2 (1·19) | 0·06 (0·59) | 1 (0·53) | 0·01 (0·07) | 0·207 |
| GP Nurse home consultation | 16 (8·94) | 1·13 (5·56) | 18 (9·78) | 1·79 (12·32) | 0·51 | 3 (1·79) | 0·04 (0·29) | 12 (6.32)) | 1·44 (9·82) | 0·065 |
| District Nurse | 1 (0·56) | 0·02 (0·22) | 5 (2·72) | 0·23 (1·74) | 0·107 | 1 (0·6) | 0·14 (1·85) | 2 (1·05) | 0·04 (0·46) | 0·469 |
| Social Worker | 12 (6·7) | 0·14 (0·73) | 11 (5·98) | 0·12 (0·52) | 0·763 | 3 (1·79) | 0·07 (0·64) | 13 (6·84) | 0·21 (1·19) | 0·159 |
| Physiotherapist | 39 (21·79) | 1·87 (5·25) | 25 (13·59) | 1·72 (8·41) | 0·846 | 17 (10·12) | 0·53 (2·16) | 20 (10·53) | 1·28 (6·5) | 0·155 |
| Occupational Therapist | 23 (12.85) | 0.67 (2.78) | 14 (7.61) | 0.92 (8.42) | 0.708 | 9 (5.36) | 0.36 (2.74) | 17 (8.95) | 0.32 (1.66) | 0.861 |
| Dietician | 3 (1.68) | 0.04 (0.32) | 2 (1.09) | 0.04 (0.43) | 0.913 | - | - | - | - | - |
| Nurse Specialist | 2 (1.12) | 0.21 (2.62) | 1 (0.54) | 0.02 (0.22) | 0.314 | - | - | - | - | - |
| Rapid Response | 4 (2.23) | 0.02 (0.15) | 4 (2.17) | 0.12 (1.41) | 0.36 | 3 (1.79) | 0.02 (0.13) | 3 (1.58) | 0.02 (0.18) | 0.848 |
| Psychotherapy/ counselling | 0 (0) | 0 (0) | 2 (1.09) | 0.02 (0.16) | 0.186 | 1 (0.6) | 0.01 (0.08) | 1 (0.53) | 0.24 (3.26) | 0.36 |
| Day centre | - | - | - | - | - | 3 (1.79) | 0.17 (1.52) | 1 (0.53) | 0.05 (0.73) | 0.357 |

a sample sizes based on all patients with available data.

Table S5 Care service use from baseline until 12 months by group. Values are number (percentages) of patients using the service and mean (SD) use.^a^

| **Service** | **Discharge – 6 months** | | | | | **6– 12 months** | | | | |
| --- | --- | --- | --- | --- | --- | --- | --- | --- | --- | --- |
|  | **Simvastatin (n=179)** | | **Placebo (n=184)** | |  | **Simvastatin (n=168)** | | **Placebo (n=190)** | |  |
|  | **Number (%)** | **Mean (SD)** | **Number (%)** | **Mean (SD)** | **p-value** | **Number (%)** | **Mean (SD)** | **Number (%)** | **Mean (SD)** | **p-value** |
| Home Help visits | 5 (2·79) | 2·46 (16·16) | 3 (1.63) | 1·94 (15·53) | 0·756 | 0 (0) | 0 (0) | 3 (1·58) | 4·93 (54·39) | 0·241 |
| Meals on Wheels visits | 1 (0·56) | 0·77 (10·31) | 0 (0) | 0 (0) | 0·311 | 0 (0) | 0 (0) | 3 (1·58) | 2·19 (17·81) | 0·112 |
| Carer visits (paid by NHS) | 10 (5·59) | 7·52 (32·76) | 10 (5·43) | 13·05 (74·53) | 0·363 | 6 (3·57) | 5·42 (29·41) | 14 (7·37) | 24·46 (104·05) | 0·023 |
| Residential Care Home (days) | 2 (1·12) | 0·08 (1·05) | 0 (0) | 0 (0) | 0·311 | 1 (0·6) | 1·07 (13·89) | 0 (0) | 0 (0) | 0·288 |
| Nursing Home (days) | 4 (2·23) | 1·36 (11·92) | 4 (2·17) | 0·62 (4·23) | 0·427 | 1 (0·6) | 1·07 (13·89) | 0 (0) | 0 (0) | 0·288 |
| Shelter (days) | 1 (0·56) | 0·04 (0·52) | 2 (1·09) | 2·63 (26·94) | 0·199 | 1 (0·6) | 0 (0) | 3 (1·58) | 1·89 (18·42) | 0·183 |
| Respite Care (days) | 2 (1·12) | 0·3 (2·86) | 1 (0·54) | 0·15 (2·06) | 0·568 | 0 (0) | 0 (0) | 1 (0·53) | 0·07 (1·02) | 0·348 |

a sample sizes based on all patients with available data.

Table S6 Health services costs (£UK) over the study period by group.^a^

| **Service Costs** | **Simvastatin**  **n=** | | **Placebo** | | **Difference (95% CI)^b^**  **Intervention -Control** |
| --- | --- | --- | --- | --- | --- |
|  | **n** | **Mean (SD)** | **n** | **Mean (SD)** |  |
| **Baseline to 6 months** | | | | | |
| Primary admission | 257 | 26,017·93 (19825·31) | 275 | 26,311·24 (20162·46) | -293·31 (-3653·94, 3161·23) |
| Other hospital services | 179 | 738·19 (2517·87) | 184 | 795·51 (3926·37) | -57·32 (-820·81, 573·50) |
| Community health Services | 179 | 395·58 (776·44) | 184 | 421·30 (1085·47) | -25·72 (-232·82, 158·40) |
| Care related services | 179 | 304·29 (1164·08) | 184 | 477·75 (2150·14) | -173·46 (-559·12, 181·97) |
| **6 to 12 months** | | | | | |
| Other hospital services | 168 | 579.49 (2215.12) | 190 | 603.51 (3211.87) | -24.02 ( -669.03, 519.74) |
| Community health services | 168 | 223.70 (682.05) | 190 | 284.46 ( 671.16) | -60.76 ( -207.01, 80.69) |
| Care related services | 168 | 245.76 (1481.22) | 190 | 639.32 (2810.66) | -393.56 (-870.93, 36.91) |
| **Total baseline-6 month^c^** | 179 | 25,228.57 (16640.52) | 183 | 27,989.23 (21313.84) | -2760.53 (-6694.82, 1186.83) |
| **Total 7-12 month** | 168 | 1048.94 (3282.70) | 190 | 1527.28 (5197.55) | -478.34 (-1472.50, 377.68) |
| **Total 12 month health service costs** | 147 | 24,183.96 (16,969.87) | 160 | 29,171.08 (24,967.77) | -4987.12 (-10059.91, -390.40) |

a sample sizes based on all patients with available data.

b Confidence interval based on 1000 bootstrap resamples. Significance is judged when the confidence interval does not cross zero. Negative costs reflected cost-savings in favour of the intervention.

c. Total costs were calculated only for patients with complete data on each of the cost components at each time point.

SD; standard deviation
