# Supplementary material for: Simvastatin for patients with acute respiratory distress syndrome: long-term outcomes and cost-effectiveness from a randomised controlled trial
Source: Crit Care. 2017 May 17;21:108. doi: 10.1186/s13054-017-1695-0 (PMC5434552; doi:10.1186/s13054-017-1695-0)
Supplement: Supplementary file 4 — HARP-2 Study Group. (DOCX 22 kb) [file 13054_2017_1695_MOESM4_ESM.docx]

**Additional File 4:** HARP-2 Study Group

**Simvastatin for patients with Acute Respiratory Distress Syndrome: long term outcomes and cost-effectiveness from a randomised controlled trial**

A. Agus Ph.D., C. Hulme Ph.D.^.^, R.M. Verghis M.Sc., C. McDowell M.Sc.^.^, C. Jackson, C.M. O’Kane Ph.D., J.G. Laffey M.D., D.F. McAuley M.D.

**HARP-2 Study Group: (* denotes Principal Investigator)**

Addenbrooke’s Hospital, Cambridge: Andrew J. Johnston*, Archana Paikray, Cat Yates, Petra Polgarova, Esther Price, Amy McInerney, Katarzyna Zamoscik

Aintree University Hospital, Liverpool: Dr Ged Dempsey*, Colette Seasman

Altnagelvin Area Hospital, Londonderry: Lynn Gilfeather*, Noel Hemmings, Sinead O’Kane

Antrim Area Hospital, Antrim: Dr Paul Johnston*, Dr Lukas Pokorny, Dr Chris Nutt, Orla O’Neill

Arrowe Park Hospital, Wirral: Prashast Prashast*, Chris Smalley, Reni Jacob

Beaumont Hospital, Dublin: Dr James O’Rourke*, Dr Syed Farjad Sultan, Carole Schilling

Birmingham Heartlands Hospital, Birmingham: Gavin D Perkins*, Teresa Melody, Keith Couper, Ron Daniels, Fang Gao, Julian Hull

Bristol Royal Infirmary, Bristol: Dr Timothy Gould*, Dr Matthew Thomas, Katie Sweet

Cork University Hospital, Cork: Dorothy Breen*, Emer Neau

Dumfries and Galloway Royal Infirmary, Dumfries: Dr Willis J. Peel*, Catherine Jardine, Dr Paul Jefferson

Freeman Hospital, Newcastle upon Tyne: Stephen E. Wright*, Kayla Harris

Frenchay Hospital, Bristol: Matthew Thomas*, Sarah Hierons

Galway University Hospital, Galway: John Laffey*, Veronica McInerney

Good Hope Hospital, Birmingham:

Guys’ and St Thomas’ Hospital, London: Luigi Camporota*, Katie Lei

Harefield Hospital, Harefield: Dr Sundeep Kaul*, Molly Chivburi

Hull Royal Infirmary, Hull: Andrew Gratix*, Rachael Bennett, Victoria Martinson, Lisa Sleight, Neil Smith

King’s College Hospital, London: Philip A. Hopkins*, Daniel Hadfield, Sarah Casboult, Fiona Wade-Smith, Julie Dawson, Clare Mellis, Clair Harris, Georgina Parsons, Sinead Helyar

Leeds Teaching Hospitals NHS Trust, Leeds: Andrew R. Bodenham*, Stuart Elliott, Zoe Beardow, Sian Birch

Mater Misericordiae University Hospital, Dublin: Brian Marsh*, Teresa Martin

Norfolk and Norwich University Hospitals, Norwich: Akesh Dhrampal*, Melissa Rosbergen

Papworth Hospital, Cambridge: Dr Stephen Webb*, Fiona Bottrill

Poole Hospital, Poole: Dr Henrik Reschreiter*, Helena Barcraft-Barnes, Julie Camsooksai

Queen Elizabeth Hospital Birmingham, Birmingham: Andrew Johnston*, Aisling Clarkson, Conor Bentley, Lauren Cooper, Yongyan Qui, Natalie Mitchell, Ronald Carrera, Arlo Whitehouse

Royal Berkshire Hospital, Reading: Christopher M. Danbury*, Nicola Jacques, Abby Brown

Royal Derby Hospital, Derby: David Rogerson*, Craig Morris

Royal Infirmary of Edinburgh, Edinburgh: Prof Timothy Walsh*, Dr Mike Gillies, Dr Grant Price, Dr Kallirroi Kefala, Dr Neil Young, David Hope, Corrienne McCulloch, Jean Antonelli, Pam Ramsay, Kirsty Everingham, Louise Boardman, Heidi Dawson, Fiona Pollock, Joanne Thompson

Royal Liverpool University Hospital, Liverpool: Dr Ingeborg D. Welters*, Dr Lee Poole, Dr Peter Hampshire, Dr Alison Hall, Karen Williams, Anna Walker, Laura Youds, Samantha Hendry, Victoria Waugh, Julie Patrick-Heselton, David Shaw

Royal Preston Hospital, Preston: Irfan Chaudry*, Jacqueline Baldwin

Royal Sussex County Hospital, Brighton: Stephen Drage*, Laura Ortiz-Ruiz de Gordoa

Royal Victoria Hospital, Belfast: Daniel McAuley*, Leona Bannon, Vanessa Quinn, Lia McNamee, Griania White

St George’s Hospital, London: Maurizio Cecconi*, Johannes Mellinghoff

St Vincents University Hospital, Dublin: Donal Ryan*, Alistair Nichol

The Royal Free Hospital, London: Dr Banwari Agarwal*, Paula Meale, Sarah James, Kulwant Dhadwal, Daniel Martin, Agnieszka Walecka, Stephen Ward

Ulster Hospital, Dundonald: John Trinder*, Samantha Hagan, Janice Montgomery, Catherine Leonard, Elizabeth Lemon, Tom Trinick

University Hospitals Coventry and Warwickshire, Coventry: Murthy Buddhavarapu*, Geraldine Ward, Christopher Bassford

Victoria Infirmary, Glasgow: Alan Davidson*, Kate McGuigan, Anissa Benchiheub, Naomi Hickey

Western Infirmary, Glasgow: Alexander Binning*, Steven Henderson

Whiston Hospital, Liverpool: J. A. Wood*

Worcester Royal Hospital, Worcester: Andrew J. Burtenshaw*, Dawn Kelly, Terry Martin, Jessica Thrush, Julie Wollaston, Stephen Graystone, Gavin Nicol, Gareth Sellors.
